# A database of weed plants in the European part of Russia

**DOI:** 10.3897/BDJ.8.e59176

**Published:** 2020-10-26

**Authors:** Alyona Tretyakova, Nickolay Grudanov, Pavel Kondratkov, Olga Baranova, Natalya Luneva, Yevgenia Mysnik, Gulnaz Khasanova, Sergey Yamalov, Maria Lebedeva

**Affiliations:** 1 Department of biodiversity and bioecology, Ural Federal University, Ekaterinburg, Russia Department of biodiversity and bioecology, Ural Federal University Ekaterinburg Russia; 2 Botanical Garden of the Ural Branch of the Russian Academy of Sciences, Ekaterinburg, Russia Botanical Garden of the Ural Branch of the Russian Academy of Sciences Ekaterinburg Russia; 3 Department of biology and fundamental medicine, Ural Federal University, Ekaterinburg, Russia Department of biology and fundamental medicine, Ural Federal University Ekaterinburg Russia; 4 Komarov Botanical Institute of the Russian Academy of Sciences, St. Petersburg, Russia Komarov Botanical Institute of the Russian Academy of Sciences St. Petersburg Russia; 5 All-Russian Research Institute of Plant Protection, St. Petersburg, Russia All-Russian Research Institute of Plant Protection St. Petersburg Russia; 6 Bashkir Research Institute of Agriculture, Ufa Federal Research Centre of the Russian Academy of Sciences, Ufa, Russia Bashkir Research Institute of Agriculture, Ufa Federal Research Centre of the Russian Academy of Sciences Ufa Russia; 7 South-Ural Botanical Garden-Institute, Ufa Federal Research Centre of the Russian Academy of Sciences, Ufa, Russia South-Ural Botanical Garden-Institute, Ufa Federal Research Centre of the Russian Academy of Sciences Ufa Russia

**Keywords:** dataset, occurrences, weed plants, field study, data paper

## Abstract

**Background:**

Weeds are plants that, although not specially cultivated, grow and often adapt to growing in arable lands. They form an ecological variant of flora, as a historically-formed set of species growing on cultivated soils. For the rational use of the chemical and biological crop protection products and to produce safe and high-quality food, up-to-date data on the floristic diversity of weeds and the patterns of its geographical change are required. The need for a weeds' database arises that allows many specialists to work together independently. However, the great value of any database lies not in its existence, but in the accumulation of data that can be used to analyse the factors affecting the species diversity of weeds.

**New information:**

A dataset of weed species diversity and their distribution in the European part of Russia, based on the results of the authors' own research from 1999 to 2019, has been created.

The dataset includes 24,284 observations of occurrences of weed plants, which were obtained on the basis of 2,049 relevés of segetal plant communities in seven regions of the European part of Russia. In total, the dataset includes information about 329 species of vascular plants growing in 65 farmlands: cereals, spring and winter crops, industrial crops, row crops and perennial grasses (Tretyakova et al. 2020).

## Introduction

Weeds are plants that, although not specially cultivated, are adapted to growing in arable lands (Ulyanova 2005,Baranova et al. 2018, Luneva 2018, Tretyakova et al. 2020). Typically, weeds are considered to be an undesirable element in crop agriculture. Their negative impact on crop development can be described in terms of competition for resources, reduction in productivity, increased challenges during harvesting and an overall increase in the cost of agricultural production.

According to contemporary agricultural practice, the main task is not to completely eliminate weed plants, but rather to limit their appearance, mitigate their harmful effect and maintain them at a level that does not adversely affect the productivity of cultivated plants. In this regard, approaches to weed management are changing. Emerging approaches include descriptions of weeds as a special ecological group of plants growing on arable land (Altieri and Liebman 1988, Ulyanova 1998, Liebman et al. 2001). In recent decades, some weed species have been identified as being under threat indicating a need for their conservation (Hofmeister 1992, Eggers and Zwerger 1998, Holub and Procházka 2000, Meyer et al. 2010).

An important principle of organic farming involves the limited and rational use of herbicides. This creates a need to search for additional crop management strategies for controlling weeds (Harker et al. 2005, Harker and O'Donovan 2013). The “ecological weed management” approach (Altieri and Liebman 1988, Heard et al. 2003), which may be the most sustainable form of weed control in the long term (Walsh and Powles 2014, Zelaya and Owen 2017), suggests a tolerance for low weed infestation. The basis for the development of new ecological strategies for weed control is the availability of complete data on the biological diversity and distribution of weeds in particular areas.

The systematic study of weeds in Russia should be attributed to the beginning of the 20th century by the works of A. I. Maltsev (Maltsev 1962). In 1934, the Academy of Sciences of the USSR published a summary "Weeds of the USSR" (Keller 1934), which contains detailed botanical descriptions of 1326 species of weeds and information on their biology and distribution. To date, a large amount of research has been carried out on the weed plants species composition in the north-west and the central part of Russia (Palkina 2011, Palkina 2015, Luneva et al. 2017 and others), in Siberia and the Russian Far East (Ulyanova 1985, Ulyanova 2005, Terekhina 2000) in the Cis-Urals and the Urals (Tuganayev 1984, Mirkin et al. 1985, Sleptsova and Rudakov 1985, Tuganayev and Semenova 1993,Tret’yakova 2006,Khasanova et al. 2014, Tuganayev et al. 2015, Khasanova et al. 2016, Kondratkov and Tretyakova 2018, Tretyakova and Kondratkov 2018, Kondratkov and Tretyakova 2019). In this paper, we present a dataset on the current diversity and distribution of weed plants in the European part of Russia. In total, the dataset contains 329 species of vascular plants growing in farmlands of 65 crops: cereals, spring and winter crops, industrial crops, row crops and perennial grasses. The dataset is expected to make a contribution to a deeper understanding of how biogeographic gradients of natural and anthropogenic factors determine the diversity of weed communities.

## General description

### Purpose

This paper aims to present the dataset on weed plants in the European part of Russia recently published in GBIF as a Darwin Core Archive.

**It includes**:

1. Populating the database on the biological diversity of weeds in the European part of Russia. The need for this is caused by significant changes taking place in Russian agriculture due to the replacement of collective and state farms by production cooperatives, agricultural holdings and large agro-industrial enterprises. During this time, wide-reaching changes also took place in terms of agricultural practices, farm areas and the range of cultivated crops. Thus, it became necessary to update the data on the weed species composition and distribution. In this work, we integrate data from weed research specialists operating in seven regions of Russia from 1999 to 2019.
2. Providing detailed information on the distribution of weeds in the regions of Russia and the occurrence of different types of arable lands. We suggest the dataset will give the scientific community an opportunity to reveal the driving factors that affect the diversity of weeds communities and to elicit its latitudinal and longitudinal variations, as well as the relationship between the weed species composition and cultivated crops. This is of key importance for the ability to predict the spread of weeds under different scenarios of climate change in different natural zones and will serve as a basis for comparison with data collected in the future.

### Additional information

Tretyakova A, Grudanov N, Kondratkov P, Baranova O, Luneva N, Mysnik Y, Khasanova G, Yamalov S, Lebedeva M (2020). Weed plants of the European part of Russia. Version 1.3. Federal State Autonomous Educational Institution of Higher Education «Ural Federal University named after the first President of Russia B.N.Yeltsin». Sampling event dataset https://doi.org/10.15468/epym22 accessed via GBIF.org on 2020-09-03.

## Project description

### Title

A database of weed plants in the European part of Russia

### Personnel

Alyona Tretyakova, Nickolay Grudanov, Pavel Kondratkov, Natalia Luneva, Evgenia Mysnik, Olga Baranova, Gulnaz Khasanova, Sergey Yamalov and Maria Lebedeva.

### Study area description

The studied areas are located in the northwest of Russia (Leningrad, Novgorod, Pskov and Vologda oblasts), in the Cis-Ural region and in the Urals (the Udmurt Republic, the Republic of Bashkortostan and Sverdlovsk oblast). The latitudinal gradient covers the taiga, forest-steppe and steppe natural zones.

### Design description

The study of the composition of weed species was carried out by the method of route counts, which evenly covered the entire territory of the regions. During the survey, a series of weed community relevés were identified and accurately georeferenced using GPS. The investigated farmlands were used to cultivate 65 crop species, including grain spring and winter crops, industrial crops, row crops and perennial grasses.

### Funding

This work was supported by Russian public funds (AAAA-A18-118011990151-7) in the framework of implementation of the State task on the “Vascular plants of Eurasia: taxonomy, ora, plant resources” (AAAA-A19-119031290052-1), by the Competitiveness of the Ural Federal University (Russian Federation Government Regulation no. 211, contract no. 02. A03.21.0006) and partially by the Russian Foundation for Basic Research (project nos. 17-44-020402 and 19-016-00135).

## Sampling methods

### Study extent

The dataset includes 24,284 (Table 1, Tretyakova et al. 2020) observations of the weed plants occurrence, which were obtained on the basis of 2049 relevés of segetal plant communities in seven regions of the European part of Russia. In total, the dataset includes information about 329 species of vascular plants growing in 65 farmlands with crops.

The identified species were as follows: *Allium cepa*, *A. porrum*, *Anethum graveolens*, *Apium graveolens*, *Avena sativa*, *Beta vulgaris*, *Brassica oleracea*, *B. napus*, *B. rapa*, *Cicer arietinum*, Daucus carota subsp. sativus, *Fagopyrum esculentum*, *Foeniculum vulgare*, *Helianthus annuus*, *Hordeum vulgare*, *Lactuca sativa*, *Linum usitatissimum*, *Medicago sativa*, *Panicum miliaceum*, *Petroselinum crispum*, *Pisum sativum*, *Phleum pratense*, *Raphanus sativus*, *Solanum tuberosum*, *Secale cereale*, *Sinapis alba*, Sorghum × drummondii, *Trifolium pratense*, *Triticale* × *Triticosecale*, *Triticum aestivum*, *Vicia sativa*, *Zea mays*.

### Sampling description

The study of weed plants communities of industrial, row crops and perennial grasses began with the stage of stemming and branching; for grain crops, it began with the stage of tilling and ended before harvesting. For perennial grasses, the 1st year planting was examined. For biennial row-crop and winter crops, both 1st and 2nd year plantings were examined. Neither the peculiarities of agrotechnical methods, nor the use of fertilisers, were taken into account. Weeds referred to any plants occurring in crops that did not serve the crop purpose, including other cultivated plants. Weeds of all ages were taken into account (seedlings, juvenile, immature, generative, excluding seeds), in any phenological (vegetation, budding, flowering, fruiting) or vital state (normally developed and depressed). In the Udmurt Republic, the weed survey was carried out by counting routes, during which floristic descriptions were provided. In the other six regions, the survey was carried out in 10×10 m plots, with at least three replicates. The distance between the plots was at least 500 m. The scientific names of plants were adjusted in accordance with the International Plants Names Index (http://www.ipni.org).

### Quality control

Materials were collected and treated by the specialists in the All-Russian Institute of Plant Protection, Komarov Botanical Garden, South-Ural Botanical Garden Institute, Bashkir Scientific Research Institute of Agriculture, Botanical Garden of the Ural Branch of RAS and the Ural Federal University.

### Step description

The Sampling Events dataset field names were chosen according to Darwin Core and include the following: “eventID”, “samplingProtocol”, “sampleSizeValue”, “sampleSizeUnit”, “informationWithhfield”, “stateProvince”, “county”, “municipality”,“habitat”, “decimalLatitude”, “decimalLongitude”, “coordinateUncertaintyInMetres”, “geodeticDatum”, “eventDate”, “year”, “countryCode”, “country”, “language”, “institutionCode”, “rightsHolder”. The Associated Occurrences dataset field names include: “eventID”, “occurrenceID”, “occurrenceStatus”, “scientificName”, “taxonRank”, “kingdom”, “stateProvince”, “county”, “municipality”,“habitat”, “decimalLatitude”, “decimalLongitude”, “geodeticDatum”, “basisOfRecord”, “eventDate”, “year”, “recordedBy”, “countryCode”, “country”, “language”, “institutionCode”, “rightsHolder”.

In order to publish our dataset on the GBIF network, we adjusted our records to the Darwin Core specifications (Wieczorek et al. 2012).

Georeferencing was carried out using GPS with WGS84 datum. Coordinate uncertainty for all occurrences was 100 metres.

## Geographic coverage

### Description

The studies were carried out in the southeast and northwest of the European part of Russia (EPR). The studied areas are distinguished by a variety of environmental conditions primarily in terms of heat provision, water availability and range of cultivated crop types. Within the areas, sharp biogeographic gradients of natural and anthropogenic factors are traced (Table 2).

**Figure 1.**
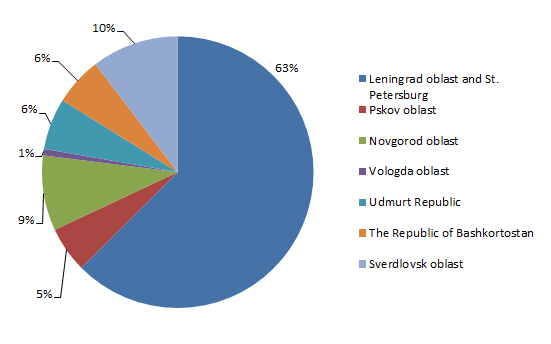
Number of occurrences in the studied regions.

In the northwest, the oblasts of Leningrad, Pskov, Novgorod and Vologda oblasts are located. This territory lies within the East European Plain. The relief is mostly characterised by low-hills. The duration of the growing season varies from 160–170 days in the south to 110–120 days in the north. The sums of positive temperatures vary from 1760ºC (north) to 2050ºC (south). The value of the hydrothermal coefficient varies from 1.7 to 1.8. In the north, podzolic soils, poor in humus and having a significantly acid pH, are widespread. The middle and south taiga spruce forest of Central European type and broad-leaved forest in the west in the presence of ash and oak is typical for this region. The share of sown area of the total area of the region varies from 2.5% to 4% (Darinsky 2001, Lobachev 2003, sel'khozportal.rf 2016, Fick and Hijmans 2017). In the southeast of the study area, Sverdlovsk oblast, the Udmurt Republic and the Republic of Bashkortostan are located. Sverdlovsk oblast is located in the central and southern parts of the Northern Urals, as well as adjacent parts of the West Siberian and East European plains. The climate of the southeast part of EPR is continental. The annual precipitation decreases from north to south and from west to east. The duration of the growing season varies from 160–170 days (in the west and south) to 110–120 days (in the mountain area of the Urals). The sums of positive temperatures vary from 1800°C (in the north) to 2300°C (in the south). The hydrothermal coefficient varies from 0.85 to 1.8 (Tuganaev 2000, Yaparov 2005, Knyazev et al. 2016, sel'khozportal.rf 2016, Fick and Hijmans 2017). Most of the study area is located in the taiga zone, where podzolic, sod-podzolic soils and grey forest soils are most widespread. In the steppe and forest-steppe zones, leached and podzolised chernozems, as well as meadow chernozem soils, are represented. The share of sown area of the total area of the region varies from 5% for the Sverdlovsk oblast to 25% in the Udmurt Republic and the Republic of Bashkortostan. The study of weed species composition was carried out in seven regions. The largest number of occurrences (15164 or 63%) were made in Leningrad oblast, while the fewest occurrences (188 or 0.8%) were made in Vologda oblast (Fig. 1).

### Coordinates

51.76 and 61.1 Latitude; 27.66 and 63.7 Longitude.

## Taxonomic coverage

### Description

The dataset includes records on weed species belonging to two plant groups (Equisetophyta and Magnoliophyta), 38 families, 182 genera and 329 species. The largest number of weed species (241) was recorded in Leningrad oblast. In other areas, the weed species diversity varied from 110 to 130 species. In Vologda oblast and the Republic of Bashkortostan, an extremely low number of weed species was noted.

The Equisetophyta group was represented by one family Equisetaceae Rich. ex DC. in which there was one genus Equisetum L. and 3 species (about 1% of all the occurrences). The Magnoliophyta group contained most occurrences (Table 3). The largest number of species (210) were drawn from the families Asteraceae, Poaceae, Fabaceae, Brassicaceae, Caryophyllaceae, Lamiaceae, Polygonaceae and Amaranthaceae; this was also reflected in the proportion of occurrences comprising 79% of the total.

The families Plantaginaceae, Boraginaceae, Rosaceae and Apiaceae are with many species (10–13 species), but a few occurrences (from 160 to 650).

Twelve families contained a few occurrences (less than 20). The families Alismataceae, Amaryllidaceae, Apocynaceae, Cyperaceae and Linaceae were represented by only one species and one occurrence.

### Taxa included

**Table:** 

Rank	Scientific Name	
phylum	EQUISETOPHYTA	
phylum	MAGNOLIOPHYTA	
class	Equisetopsida	
class	Liliopsida (Monocotyledones)	
class	Magnoliopsida (Dicotyledones)	

## Traits coverage

### Data coverage of traits

PLEASE FILL IN TRAIT INFORMATION HERE

## Temporal coverage

### Notes

24 June 1999 – 30 July 2019.

**Figure 2.**
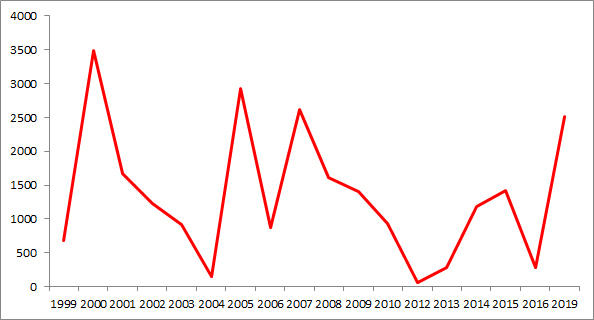
Number of occurrences in temporal scope.

The presented database contained information about weeds occurrences from 1999 till 2019. Most weed occurrences were made in 2000, 2005, 2007 and 2019. Fewer occurrences were made in 1999, 2003, 2004, 2006, 2010, 2012 and 2016 (Fig. 2).

## Usage licence

### Usage licence

Creative Commons Public Domain Waiver (CC-Zero)

### IP rights notes

This work is licensed under a Creative Commons Attribution (CC-BY) 4.0 License.

## Data resources

### Data package title

Weed plants of the European part of Russia

### Resource link

https://www.gbif.org/dataset/edd76a7a-64e0-4008-a741-105ecd67e339

### Alternative identifiers

https://doi.org/10.15468/epym22

### Number of data sets

2

### Data set 1.

#### Data set name

Darwin Core Archive Event dataset

#### Data format

Darwin Core

#### Number of columns

20

#### Character set

UTF-8

#### Download URL

https://www.gbif.org/dataset/edd76a7a-64e0-4008-a741-105ecd67e339

#### Data format version

1.3

#### Description

Data on the weed plants species diversity in the European part of Russia are presented. The dataset includes two tables in Darwin Core format: Sampling Events with 20 fields and about 2049 records and Associated Occurrence with 23 fields and about 24284 records. The weed plants refer to the plants that are not specially cultivated, but adapted to grow in arable areas and reduce the crops size and quality. The dataset was compiled from the authors' own research from 1999 to 2019. Herbarium samples are stored in the herbarium collections of the Ural Federal University (UFU), the All-Russian Institute for Plant Protection, Botanical Institute named after V. L. Komarov (LE), Udmurt State University (UDU) and the South Ural Botanical Garden Institute. The dataset contains 2049 sampling events, which include 24,284 observations of the weed plants' occurrence (associated occurrences) in arable lands in the EPR. The dataset includes 330 species of vascular plants growing in 60 cultivated crops: spring and winter crops, industrial crops, row crops and perennial grasses. This dataset is the first and most important step in summarising the information on the current diversity and geographical distribution of weed plants in the EPR.

**Data set 1.** 

Column label	Column description
eventID	An identifier of a particular event http://rs.tdwg.org/dwc/terms/eventID
samplingProtocol	The name of, reference to, or description of the method or protocol used during an Event. Included value: vegetation releve. http://rs.tdwg.org/dwc/terms/samplingProtocol
sampleSizeValue	A numeric value for a measurement of the size (time duration, length, area or volume) of a sample in a sampling event. http://rs.tdwg.org/dwc/terms/sampleSizeValue
sampleSizeUnit	The unit of measurement of the size (time duration, length, area or volume) of a sample in a sampling event. http://rs.tdwg.org/dwc/terms/sampleSizeUnit
informationWithheld	Additional information that exists, but that has not been shared in the given record. Included value: species abundance http://rs.tdwg.org/dwc/terms/informationWithheld
stateProvince	The name of the next smaller administrative region than country. http://rs.tdwg.org/dwc/terms/stateProvince
county	The full, unabbreviated name of the next smaller administrative region than stateProvince. http://rs.tdwg.org/dwc/terms/county
municipality	The full, unabbreviated name of the next smaller administrative region than county. http://rs.tdwg.org/dwc/terms/municipality
habitat	A category or description of the habitat in which the Event occurred. Included crops. http://rs.tdwg.org/dwc/terms/habitat
decimalLatitude	The geographic latitude (in decimal degrees, using the spatial reference system given in geodeticDatum) of the geographic centre of a Location. http://rs.tdwg.org/dwc/terms/decimalLatitude
decimalLongitude	The geographic longitude (in decimal degrees, using the spatial reference system given in geodeticDatum) of the geographic centre of a Location. http://rs.tdwg.org/dwc/terms/decimalLongitude
coordinateUncertaintyInMetres	The horizontal distance (in metres) from the given decimalLatitude and decimalLongitude describing the smallest circle containing the whole of the Location. Included value: 100. http://rs.tdwg.org/dwc/terms/coordinateUncertaintyInMeters
geodeticDatum	The ellipsoid, geodetic datum or spatial reference system (SRS) upon which the geographic coordinates given in decimalLatitude and decimalLongitude are based. http://rs.tdwg.org/dwc/terms/geodeticDatum
eventDate	The date-time or interval during which an Event occurred. http://rs.tdwg.org/dwc/terms/eventDate
year	The four-digit year in which the Event occurred, according to the Common Era Calendar. http://rs.tdwg.org/dwc/terms/year
countryCode	The standard code for the country in which the Location occurs. Included value: RU. http://rs.tdwg.org/dwc/terms/countryCode
country	The name of the country or major administrative unit in which the Location occurs. Included value: Russia http://rs.tdwg.org/dwc/terms/country
language	A language of the resource. Included value: ru. http://purl.org/dc/terms/language
institutionCode	The name (or acronym) in use by the institution having custody of the object(s) or information referred to in the record. http://rs.tdwg.org/dwc/terms/institutionCode
rightsHolder	A person or organisation owning or managing rights over the resource.http://purl.org/dc/terms/rightsHolder

### Data set 2.

#### Data set name

Darwin Core Archive Occurrence dataset

#### Data format

Darwin Core

#### Number of columns

23

#### Character set

UTF-8

#### Download URL

https://www.gbif.org/dataset/edd76a7a-64e0-4008-a741-105ecd67e339

#### Data format version

1.3

#### Description

The dataset includes a table in Darwin Core format with 23 fields and about 24284 records.

**Data set 2.** 

Column label	Column description
eventID	Event identifier. http://rs.tdwg.org/dwc/terms/eventID
occurrenceID	An identifier for the Occurrence (as opposed to a particular digital record of the occurrence). http://rs.tdwg.org/dwc/terms/occurrenceID
occurrenceStatus	A statement about the presence or absence of a Taxon at a Location. Included value: present. http://rs.tdwg.org/dwc/terms/occurrenceStatus
scientificName	The full scientific name. http://rs.tdwg.org/dwc/terms/scientificName
taxonRank	The taxonomic rank of the most specific name in the scientificName. http://rs.tdwg.org/dwc/terms/taxonRank
kingdom	The full scientific name of the kingdom in which the taxon is classified. Included value: Planta. http://rs.tdwg.org/dwc/terms/kingdom
stateProvince	The name of the next smaller administrative region than country. http://rs.tdwg.org/dwc/terms/stateProvince
county	The full, unabbreviated name of the next smaller administrative region than stateProvince. http://rs.tdwg.org/dwc/terms/county
municipality	The full, unabbreviated name of the next smaller administrative region than county. http://rs.tdwg.org/dwc/terms/municipality
habitat	A category or description of the habitat in which the Event occurred. Included various crops. http://rs.tdwg.org/dwc/terms/habitat
decimalLatitude	The geographic latitude (in decimal degrees, using the spatial reference system given in geodeticDatum) of the geographic centre of a Location. http://rs.tdwg.org/dwc/terms/decimalLatitude
decimalLongitude	The geographic longitude (in decimal degrees, using the spatial reference system given in geodeticDatum) of the geographic centre of a Location. http://rs.tdwg.org/dwc/terms/decimalLongitude
coordinateUncertaintyInMetres	The horizontal distance (in metres) from the given decimalLatitude and decimalLongitude describing the smallest circle containing the whole of the Location. Included value: 100. http://rs.tdwg.org/dwc/terms/coordinateUncertaintyInMeters
geodeticDatum	The ellipsoid, geodetic datum or spatial reference system (SRS) upon which the geographic coordinates given in decimalLatitude and decimalLongitude are based. http://rs.tdwg.org/dwc/terms/geodeticDatum
basisOfRecord	The specific nature of the data record. Included value: HumanObservation. http://rs.tdwg.org/dwc/terms/basisOfRecord
eventDate	The date-time or interval during which an Event occurred. http://rs.tdwg.org/dwc/terms/eventDate
year	The four-digit year in which the Event occurred, according to the Common Era Calendar. http://rs.tdwg.org/dwc/terms/year
recordedBy	A list (concatenated and separated) of names of people, groups or organisations responsible for recording the original Occurrence. http://rs.tdwg.org/dwc/terms/recordedBy
countryCode	The standard code for the country in which the Location occurs. Included value: RU. http://rs.tdwg.org/dwc/terms/countryCode
country	The name of the country or major administrative unit in which the Location occurs. Included value: Russia http://rs.tdwg.org/dwc/terms/country
language	A language of the resource. Included value: ru. http://purl.org/dc/terms/language
institutionCode	The name (or acronym) in use by the institution having custody of the object(s) or information referred to in the record. http://rs.tdwg.org/dwc/terms/institutionCode
rightsHolder	A person or organisation owning or managing rights over the resource. http://purl.org/dc/terms/rightsHolder

## Figures and Tables

**Table 1.** Number of records made by authors.

**Authors**	**Region**	**Number of records**	**Number of species**
Luneva N., Mysnik E.	Leningrad oblast and Saint Petersburg	15,164	241
Luneva N., Mysnik E.	Pskov oblast	1,320	126
Luneva N., Mysnik E.	Novgorod oblast	2,170	133
Luneva N., Mysnik E.	Vologda oblast	188	55
Baranova O.	Udmurt Republic	1,531	199
Khasanova G., Yamalov S., Lebedeva M.	Republic of Bashkortostan	1,397	24
Tretyakova A., Grudanov N., Kondratkov P.	Sverdlovsk oblast	2,514	111
Total		24,284	329

**Table 2.** Geographical characteristics of the studied regions (sel'khozportal.rf 2016, Fick and Hijmans 2017, GKS.ru 2018).

**The main parameters**	**North-west**			**South-east**			
	**LR**	**PO**	**NR**	**VR**	**UR**	**RB**	**SR**
North latitude	58°31′–59°20′	55°43′–57°14′	57°0′–59°18′	58°39′–60°8′	55°40'–60°30'	51°31′–56°34′	56º03' –61º57'
East longitude	28°20′–29°36′	27°39′–30°24′	29°57′–35°45′	35°13′–46°42′	48°20'–56°40'	53°10′– 59°59′	57º14'–66º11'
Mean air temperature, °C	4	5	4	2	3	3	2
Annual precipitation, mm	654	600	653	607	580	522	534
Region area, km^2^	83,9	55,4	54,5	144,5	42,1	142,9	194,3
Population, thousand of people	1,813	666	606	1,176	1,513	4,063	4,325
Population density, people/km^2^	21,6	12.0	11.1	8.1	35.9	28.4	22.3
Crop area, km^2^	2,299	2,453	1,785	3,724	10,289	36,367	8,988
The share of crop area in the total area of the region, %	2.7	4.4	3.3	2.6	24.5	25.4	4.6
The sum of temperatures above 10 °C							
on the northern border of the region	1,848	1,900	2,052	1,813	1,889	2,068	1,380
on the southern border of the region	2,041	2,068	2,063	1,967	2,301	2,393	1,985
Hydrothermal coefficient							
on the northern border of the region	1.83	1.82	1.75	1.71	1.,59	1.43	2.02
on the southern border of the region	1.75	1,75	1.82	1.68	1.12	0.85	1.49
Average height m a.s.l.	109	110	120	162	180	435	513
Average temperature in January, °С	–9…–11	–8...–10	–8…–10	–10…–11	–15	–15…–17	–18
Average temperature in July, °С	+16…+17	+17	+16…+18	+16…+17	+17	+17…+19	+17
Duration of the growing season, days	205–220	125–150	175	105–120	190–200	200–205	170
Natural zone	middle and south taiga	south taiga, temperate forest	south taiga, temperate forest	middle and south taiga	south taiga, temperate forest	temperate and broadleaved forest, forest steppe, steppe	middle and south taiga, forest steppe

Note (hereinafter, the symbol of the regions): Region: LO – Leningrad oblast; PO – Pskov oblast, NR – Novgorod oblast; VR – Vologda oblast; UR – Udmurt Republic; RB – Republic of Bashkortostan; SR – Sverdlovsk oblast.

**Table 3.** Taxonomic distribution of weed species and species occurrences amongst families in the dataset. Families are listed in order of decreasing total number of occurrences.

Plant family	Number of			
	genera	species	entries	% of all occurrences
Asteraceae Bercht. & J.Presl	35	57	6,232	25.7
Brassicaceae Burnett	17	25	2,571	10.6
Polygonaceae Juss.	5	15	2,380	9.8
Poaceae Barnhart	20	33	1,798	7.4
Lamiaceae Martinov	10	16	1,797	7.4
Caryophyllaceae Juss.	11	19	1,780	7.3
Amaranthaceae Juss.	7	14	1,486	6.1
Fabaceae Lindl.	9	31	1,062	4.4
Plantaginaceae Juss.	4	10	649	2.7
Violaceae Batsch	1	2	606	2.5
Rubiaceae Juss.	1	6	566	2.3
Boraginaceae Juss.	9	12	510	2.1
Papaveraceae Juss.	2	2	503	2.1
Ranunculaceae Juss.	3	8	405	1.7
Equisetaceae Michx.ex DC.	1	3	317	1.3
Geraniaceae Juss.	2	3	285	1.2
Rosaceae Juss.	3	10	241	0.9
Convolvulaceae Juss.	2	2	193	0.8
Euphorbiaceae Juss.	1	2	187	0.8
Urticaceae Juss.	1	2	183	0.8
Apiaceae Lindl.	12	13	165	0.7
Solanaceae Juss.	3	6	69	0.3
Campanulaceae Juss.	1	3	58	0.2
Onagraceae Juss.	1	4	51	0.2
Hypericaceae Juss.	1	2	33	0.1
Cannabaceae Martinov	1	1	27	0.1
Malvaceae Juss.	2	3	19	< 0.1
Primulaceae Batsch ex Borkh.	2	3	15	< 0.1
Caprifoliaceae Juss.	2	2	13	< 0.1
Juncaceae Juss.	2	5	11	< 0.1
Orobanchaceae Vent.	3	5	8	< 0.1
Scrophulariaceae Juss.	2	4	6	< 0.1
Sapindaceae Juss.	1	1	3	< 0.1
Alismataceae Vent.	1	1	1	< 0.1
Amaryllidaceae	1	1	1	< 0.1
Apocynaceae Juss.	1	1	1	< 0.1
Cyperaceae Juss.	1	1	1	< 0.1
Linaceae DC. ex Perleb	1	1	1	< 0.1
Total				
38	182	329	24234	100
